# A novel homozygous *TSGA10* missense variant causes acephalic spermatozoa syndrome in a Pakistani family

**DOI:** 10.1186/s12610-024-00220-7

**Published:** 2024-02-05

**Authors:** Khalid Khan, Xiangjun Zhang, Sobia Dil, Ihsan Khan, Ahsanullah Unar, Jingwei Ye, Aurang Zeb, Muhammad Zubair, Wasim Shah, Huan Zhang, Muzammil Ahmad Khan, Limin Wu, Bo Xu, Hui Ma, Zina Wen, Qinghua Shi

**Affiliations:** 1 Division of Reproduction and Genetics, The First Affiliated Hospital of University of Science and Technology of China, Institute of Health and Medicine, Hefei Comprehensive National Science Center, School of Basic Medical Sciences, Division of Life Sciences and Medicine, Biomedical Sciences and Health Laboratory of Anhui Province, University of Science and Technology of China, Hefei, 230027 China; 2 Gomal Centre of Biochemistry and Biotechnology, Gomal University, Dera Ismail Khan, Khyber Pakhtunkhwa, Pakistan; 3 Chengdu Xi’nan Gynecological Hospital, Chengdu, Sichuan China

**Keywords:** Infertility, *TSGA10*, Acephalic spermatozoa, Missense variations

## Abstract

**Background:**

Acephalic spermatozoa syndrome is a rare type of teratozoospermia causing male infertility due to detachment of the sperm head and flagellum, which precludes fertilization potential. Although loss-of-function variations in several genes, including *TSGA10,* have been associated with acephalic spermatozoa syndrome, the genetic cause of many cases remains unclear.

**Results:**

We recruited a Pakistani family with two infertile brothers who suffered from acephalic spermatozoa syndrome. Through whole-exome sequencing (WES) followed by Sanger sequencing, we identified a novel missense variant in *TSGA10* (c.1112T > C, p. Leu371Pro), which recessively co-segregated with the acephalic spermatozoa syndrome within this family. Ultrastructural analyses of spermatozoa from the patient revealed that 98% of flagellar cross-sections displayed abnormal axonemal ultrastructure, in addition to the head-flagellum detachment. Real-time quantitative PCR analysis revealed almost no detectable *TSAG10* mRNA and western blot analysis also failed to detect TSAG10 protein in patient's sperm samples while TSGA10 expression was clearly detected in control samples. Consistently, immunofluorescence analysis demonstrated the presence of TSGA10 signal in the midpiece of sperm from the control but a complete absence of *TSGA10* signal in sperm from the patient.

**Conclusion:**

Altogether, our study identifies a novel *TSGA10* pathogenic variant as a cause of acephalic spermatozoa syndrome in this family and provides information regarding the clinical manifestations associated with *TSGA10* variants in human.

**Supplementary Information:**

The online version contains supplementary material available at 10.1186/s12610-024-00220-7.

## Résumé

**Contexte:**

Le syndrome des spermatozoïdes acéphaliques est un type rare de tératozoospermie provoquant une infertilité masculine en raison du détachement de la tête et du flagelle des spermatozoïdes, ce qui exclut une potentielle fécondation. Bien que des variations de perte de fonction dans plusieurs gènes, y compris TSGA10, aient été associées au syndrome des spermatozoïdes acéphaliques, la cause génétique de nombreux cas reste incertaine.

**Résultats:**

Nous avons recruté une famille pakistanaise avec deux frères infertiles qui souffraient du syndrome des spermatozoïdes acéphaliques. Grâce au séquençage de l’exome entier (WES) suivi du séquençage Sanger, nous avons identifié un nouveau variant faux-sens dans TSGA10 (c.1112T > C, p. Leu371Pro), qui co-ségréguait de manière récessive avec le syndrome des spermatozoïdes acéphaliques au sein de cette famille. Les analyses ultrastructurales des spermatozoïdes des patients ont révélé que 98% des coupes transversales flagellaires présentaient une ultrastructure axonémiques anormales, en plus du décollement tête-flagelle. L’analyse quantitative par PCR en temps réel n’a révélé presque aucun ARNm TSAG10 détectable; l’analyse par transfert Western n’a pas non plus réussi à détecter la protéine TSAG10 dans les échantillons de sperme des patients, tandis que l’expression de TSGA10 a été clairement détectée dans les échantillons du témoin. De manière cohérente, l’analyse par immunofluorescence a démontré la présence du signal TSGA10 dans la partie médiane des spermatozoïdes du témoin, mais une absence totale de signal TSGA10 chez ceux des patients.

**Conclusion:**

Dans l’ensemble, notre étude identifie un nouveau variant pathogène de TSGA10 comme cause du syndrome des spermatozoïdes acéphaliques dans cette famille et fournit des informations concernant les manifestations cliniques associées aux variants de TSGA10 chez l’homme.

**Mots-clés:**

Infertilité, TSGA10, Spermatozoïdes acéphaliques, Variations faux-sens.

## Introduction

Infertility is the third major disease impacting human health in the twenty-first century according to the World Health Organization [1]. Males are responsible for almost 50% of infertility [2], whereas acephalic spermatozoa syndrome (ASS) is one of the severest disorders in male infertility. ASS is characterized by the presence of a lot of headless sperm tails, a few tailless sperm heads and sperm with aberrant head–tail link [3]. Occasionally, acephalic spermatozoa have been found to be familial, implying that it is a genetic condition [4–7]. However, the specific pathophysiology is still unknown.

Several genes have been shown to play an essential role in development of acephalic spermatozoa [8]. *SUN5* mutations were reported to account for acephalic spermatozoa in one-third to one-half of reported patients with ASS [9–12]. Another study reported that the homozygous p.G928D mutation in *BRDT* (Bromodomain Testis Associated gene) leads to acephalic spermatozoa in a consanguineous family [5]. In addition, *PMFBP1* mutations were also associated with acephalic spermatozoa in infertile human patients, which was functionally validated by using a knockout mouse model [11, 13]. However, the causes of ASS in many patients remain uncharacterized.

*TSGA10* (Testis-specific gene 10) is a testis-specifically expressed gene, encoding a protein highly expressed in the sperm. The TSGA10 protein can be cleaved into two parts: a 27-kDa N-terminus found in the principal component, and a 55-kDa C-terminus found in the centrosome and basal body [14–17], which is a centrosome scaffold component associated with mother centrioles. The 55-kDa C-terminus of *TSGA10* can interact with ODF2 [16], implying that the TSGA10 C-terminus is involved in centriole assembly and function, particularly in the head–tail link. Previously, loss of function mutation in *TSGA10* was reported to be associated with ASS in infertile patients [18]. Additionally, male mice heterozygous for *Tsga10* deletion were also found to be infertile and presented significantly reduced sperm motility because of disordered mitochondrial sheath formation [19]. Hence, the genetic and phenotype correlation between the *TSGA10* mutation and ASS are still need to be confirmed.

In the present study, a Pakistani family who suffered from ASS was enrolled in this research, and a novel *TSGA10* variant was identified by using whole exome sequencing (WES) and Sanger sequencing analysis. Further, TEM examination of spermatozoa from *TSGA10* mutant patient revealed headless spermatozoa, disrupted ODF and disorganized axonemal structure, which is in consistent with the reported phenotype of *TSGA10* mutant patient [18]. Overall, all these findings provide the genetic evidence that the homozygous missense variant in *TSGA10* is pathogenic for acephalic spermatozoa syndrome.

## Materials and methods

### Ethical statement

This family was recruited from the local hospital in Khyber Pakhtunkhwa, Pakistan, and recorded in the Human Reproductive Disease Resource Bank at the University of Science and Technology of China’s (USTC). At the beginning of this study a detailed written consent forms from the patients and controls were signed. The Gomal University Pakistan and USTC institutional ethics committees approved this study.

### Patients and medical examinations

In this study we recruited a Pakistani family with two infertile brothers (35- and 29- years-old respectively). The patients had no previous exposure to hazardous chemicals and did not drink alcohol or smoke. They had no history of urogenital or other reproductive diseases. The patients had normal erection and ejaculation according to the clinical examination. Sperm concentration and motility were conducted from patient, and sperm smear slides were prepared according to the instructions of World Health Organization [20].

### HE staining of *TSGA10* patient’s spermatozoa

In accordance with WHO standards [20], the patient III:2 had undergone routine semen analysis twice. Semen smears slides were prepared and sequentially immersed in 4% paraformaldehyde (PFA) for 5 min, washed with 1X phosphate buffered saline (PBS) twice for 5 min each, stained in hematoxylin (Solarbio, Beijing, China) for 30 min, dipped in purified water three times, immersed in 50% acidic ethanol, and kept in tap water for 2 min. The slides were then dehydrated in 50% and 80% ethanol for 5 min each, stained for 5 min with Eosin Azure (Solar bio), serially dehydrated twice in 100% ethanol for 5 min each and in xylene for 5 min, and eventually covered with coverslips and natural balsam. At least 200 spermatozoa were captured and analyzed under the optical microscope (Nikon, Tokyo, Japan).

### WES and variant filtration

Blood samples were taken from the patients (III: 1 and III: 2), their fertile brother (III: 3), and their mother (II: 2). WES was carried out as described previously [21]. Variants were filtered using the following criteria: (a) variants with autosomal recessive inheritance pattern were included; (b) variants in linkage regions with logarithm odds scores > 0.01 were included. (c) variants having minor allele frequencies less than 0.05 [22] in any public database, including the 1000 Genomes Project, ESP6500, or gnomAD database, as well as homozygous variants in our in-house WES database compiled from 578 fertile men (41 Pakistani, 254 Chinese, and 283 European) were included; (d) protein sequence-altering variants (nonsense, missense, splice-site, and coding indels) were included; (f) variants in genes expressed in the testis were included; (g) variants predicted to be deleterious by more than half of the software (provided by ANNOVAR) covering them were included [23], and (h) variants within genes dispensable for spermatogenesis in mice were excluded based on spermatogenesis online 1.0 or literature searches [24]. Sanger sequencing was further used to confirm the filtered variants in all of the available family members (Supplementary Fig. 1). Lists of the primer sequences used for Sanger sequencing are given in Supplementary Table 2.

### Real time quantitative PCR

Total RNA for qPCR was extracted from semen samples and stored in Trizol reagents (TakaRa Bio) for the patient and control samples. For cDNA synthesis, 1 μg RNA was reversely transcribed using PrimSript RT Reagent Kit (Takara) according to the manufacturer’s instructions as previously described [25]. The relative expression level of *TSGA10* mRNA was calculated by normalization of the cycle threshold (Ct) value of samples to the corresponding Ct values of *ACTB*. The primer sequences are given in Supplementary Table 1.

### Transmission electron microscopy

TEM was performed as previously described [25, 26]. Spermatozoa from the patient and control (fertile) were collected, fixed in 0.1 mol/L phosphate buffer (PB; pH 7.4), which contained 0.2% picric acid, 8% glutaraldehyde, and 4% paraformaldehyde, and then kept overnight at 4°C. Next day after washing with 0.1 mol/L PB, the samples were fixed with 1% osmium tetroxide. Dehydration of spermatozoa was done using different graded alcohol solutions (30%, 60%, 90% and 100%; 10 min for each bath), followed by inclusion in epon resin and acetone mixture. Then the samples were sliced into ultra-thin (70 nm) sections and stained with lead and uranyl acetate. The ultrastructure of spermatozoa cross-sections was captured and examined using Tecnai 10 or 12 Microscopes (Philps CM10, Philips Electronics, Eindhoven, and the Netherlands) at 120 kV or 100 kV.

### In silico analysis of *TSGA10*

The genomic sequence of *TSAG10* was retrieved from the NCBI (http//ncbi.nlm.nih.gov). To assess the deleterious effect of the variant, we used several prediction tools, including SIFT, PANTHER, PredictSNP, MAPP, SNAP, Polyphen-1 and Polyphen-2 [27–34]. In addition, we conducted multiple sequence alignment of the TSGA10 protein and analyzed the evolutionary conservation of the mutated residue across various species using Mega-X and Jalview [35–37].

### Immunofluorescence staining

Immunofluorescence staining was conducted on the spermatozoa from the patients and control individuals, as previously reported [25, 38]. Briefly, patient sperm samples were smeared onto clean slides and fixed with 4% paraformaldehyde. The slides were then washed three times with PBS. The slides were permeabilized with 0.5% Triton X-100 for 30 min and blocked with 1% BSA. Primary antibodies, namely anti-α-tubulin (Sigma, F2168), anti-TSGA10 (12,593–1-AP, Proteintech Group) were incubated with the slides overnight at 4°C. On the next day, the slides were washed with PBST (PBS containing 0.1% Triton X-100) and subsequently incubated with secondary antibodies DAR555 (Molecular Probes, A31572) and GAM488 (Molecular Probes, A21121) for 1 h at 37°C. After three washes with PBST, the slides were sealed with Hoechst and Vectashield. Image acquisition was performed using a microscope (Olympus). The information of primary and secondary antibodies used and their dilutions are provided in Supplementary Table 2.

### Western blotting

To obtain protein lysate, the semen sample was subjected to lysis using a lysis buffer (50 mM Tris–HCl (pH 7.5), 150 mM NaCl, 2.5 mM EDTA, and 0.5% Triton X-100). The mixture was then centrifuged at 4°C for 15 min, and the resulting supernatant was collected as previously described [39]. For denaturation, the supernatant was incubated in protein loading buffer (100 mM Tris–HCl (pH 7.4), 2% SDS, 15% glycerol, 0.1% bromophenol blue, and 5 mM dithiothreitol, DTT) for 10 min. Subsequently, the protein was separated through SDS-PAGE electrophoresis and transferred to a nitrocellulose filter membrane (GE Healthcare, 10,600,002, CT, USA). The membrane was blocked using a TBST solution (50 mM tris (pH 7.4), 150 mM NaCl, and 0.1% Tween-20), containing5% skimmed milk for 1 h. Primary antibodies were then incubated with the membrane overnight at 4°C. The membrane was subsequently washed three times with TBST solution for 10 min each. Following a one-hour incubation with horseradish peroxidase-coupled secondary antibodies, the membrane was developed using a chemiluminescent substrate (Thermo Fisher Scientific, 34,580) and imaged using the ImageQuant LAS 4000 Imaging System (GE Healthcare). The information of primary and secondary antibodies used and their dilutions are provided in Supplementary Table 2.

## Results

### Physical and semen characteristics of patients

**Fig. 1:**
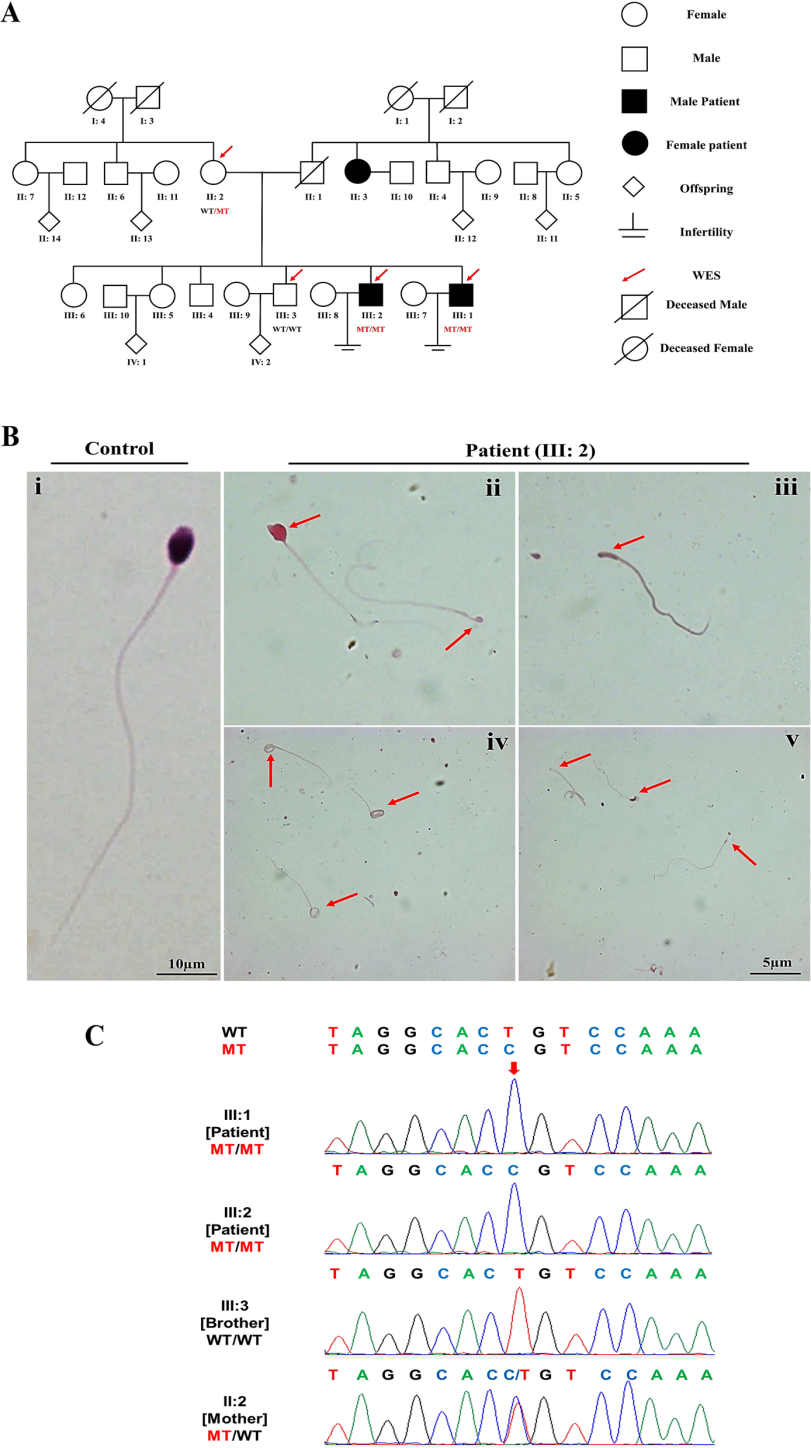
A *TSGA10* variant identified in a non-consanguineous Pakistani family with acephalic spermatozoa. **A** Pedigree of the Pakistani family with two infertile male patients, P1 (III: 1) and P2 (III: 2). Arrowheads point to the four individuals for which whole-exome sequencing (WES) was performed. Slashes denotes deceased family members. Squares represent males and circles represent females. Filled squares or circle represent male and female patient respectively. Clear symbols signify normal individuals. Diamonds represent multiple individuals without sex information. WT, wild-type. MT, (c.1112T > C, p. Leu371Pro). **B** Hematoxylin staining of the patient’s sperm. The sperm morphology was primarily acephalic. The semen smear slides were stained with the H&E staining, showing only the tails (ii-v). The red arrows indicate the acephalic spermatozoa. Scale bar: 10 μm, 5 μm respectively (**C**) Verification of the *TSGA10* variant, c.1112T > C, in genomic DNA from all available family members

In this study, a Pakistani family with two infertile brothers was recruited (Fig. 1A). Both affected individuals had normal positions of abdominal viscera. The physical characteristic of patient (III: 1 and III: 2) are summarized in Table 1. III: 1 refused to provide semen samples and only shared his previous medical records. Patients (III: 1 and III: 2) had very low sperm concentrations (1 million/ml and 0.5 million/ml, respectively). Their sperm progressive motility were 4.0% and 7.0% respectively, which were below the reference range (32%) suggested by WHO [1]. Only 5% of spermatozoa were morphologically normal. We carried out the H&E staining of semen smear slides from patient (III: 2), to find out the morphological abnormalities of the spermatozoa. Among the sperm with abnormal morphology, almost no sperm were found with normal sperm head. The most prominent defect is absence of sperm head, accounting for 31% of total sperm. Multiple morphological defects of the sperm head were also identified, such as tapered, pyriform, round, and amorphous heads (Fig. 1B) and (Table 1). These results indicate that patient (III: 2) suffered from ASS.

**Table 1:** Semen characteristics and sperm head morphology of patients homozygous for the *TSGA10* variant

-	Patient III:1	Patient III:2	Reference values
**Physical information**^a^			
Age (years)^b^	43	40	-
Marriage (years)	19	15	-
Weight (kg)	68	76	-
**Semen characteristics**^c^			
Semen volume (mL)	3.2	2.3	> 1.5
Semen pH	7	7	> 7.2
Sperm concentration (10^6^/mL)	1	0.5	> 15.0
Progressive motility (%)	4*	7*	> 32.0
Non progressive motility (%)	42	6	-
Total motility (%)	46	13*	> 40.0
Immotile (%)	54	87	-
Sperm with normal morphology	-	5	
**Sperm head morphology**			
Normal head (%)	-	0.03	-
Absent head (%)	-	31.0	-
Tapered head (%)	-	16.3	-
Pyriform head (%)	-	19.6	-
Round head (%)	-	18.3	-
Amorphous head (%)	-	14.2	-

^a^Physical examination was performed by the local andrologist

^b^Ages at the manuscript submission

^c^Semen analysis was performed for each infertile individual following the WHO guidelines (World Health Organization, 2010)

^*^Abnormal values

### WES identified a homozygous missense variant in *TSGA10*

To determine the origin of the pathogenesis in this family, genomic DNA was extracted from the whole blood samples of the patients (III: 1) and (III: 2), their mother (II: 2), and fertile brother (III: 3), followed by WES analysis. Further bioinformatics analysis was performed for screening candidate pathogenic variations. Through a series of variant filtration methods, a *TSGA10* homozygous missense variant (ENST00000393483, (c.1112T > C, p. Leu371Pro) was found (Supplementary Fig. 1). Later, Sanger sequencing confirmed that this variant was co-inherited with the ASS in this family in a recessive manner (Fig. 1C). Noticeably, we did not find any known genes related to acephalic spermatozoa except *TSGA10* in these two patients. Therefore, we focused on this *TSGA10* variant and hypothesized that this variant was potentially the cause of ASS in patients.

### In silico analysis of the *TSGA10* variant

**Fig. 2:**
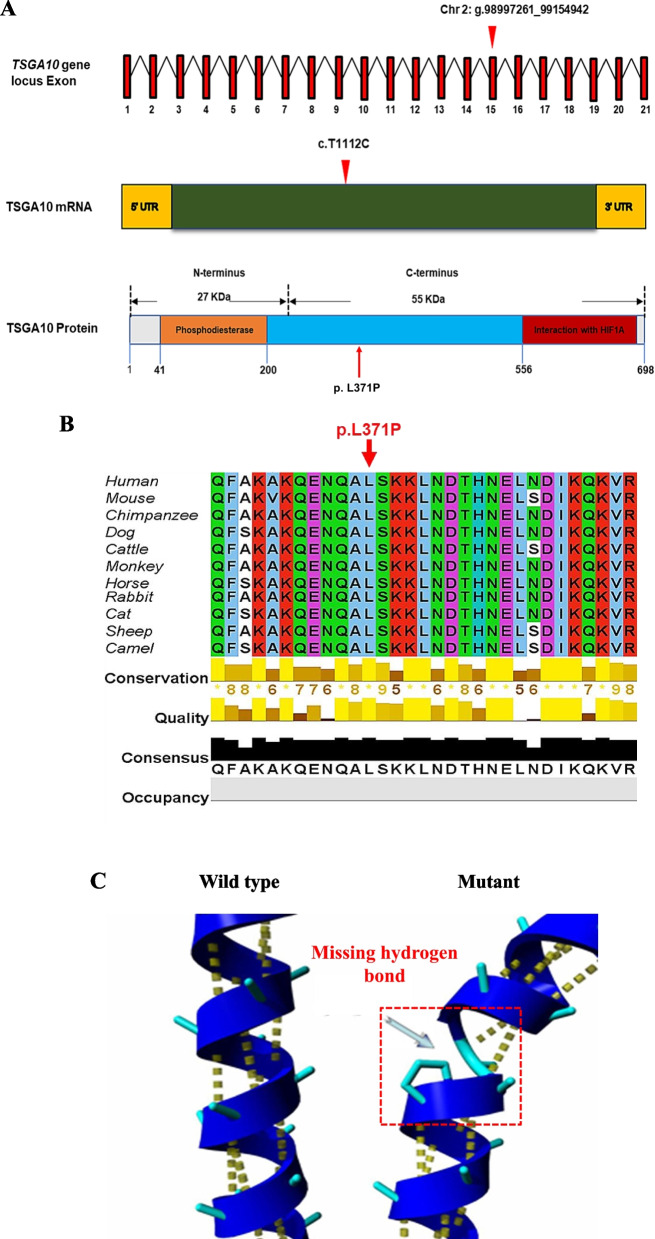
Position of the identified *TSGA10* variant at genomic, transcript and protein levels. **A** *TSGA10* is located on chromosome (chr) 2, comprises 21 exons, and encodes a predicted 698-amino-acid protein (NCBI: ENST00000393483.8; UniProt KB: Q9BZW7-1). The *TSGA10* variant (c.1112T > C, p. Leu371Pro), is located on exon 15. **B** Conservative analysis of the missense variant site in different species. **C** Effect of the variant on the helical structure of protein, predicted by Reprof software. Proline disrupts an α-helix. Due to this *TSAG10* ^p. Leu371Pro^ variant, the α-helix is disrupted, which could have severe effects on the structure of the protein

To evaluate the deleteriousness of this homozygous missense variant, we first performed several in silico analyses. This variant was identified in exon 15 of the *TSGA10* and the affected amino acid was located in the functional domain of phosphodiesterase, as illustrated in Fig. 2A. Specifically the missense variant resulted in the replacement of the residue Leucine (L) at position 371 of the TSAG10 with Proline (P). The affected leucine is highly conserved in different species, indicating its functional significance (Fig. 2B). Figure 2C illustrates the position of the leucine residue within an α-helix of the wild-type TSGA10 protein. A mutation introducing proline was predicted to disrupt this α-helix and significantly alter the protein's structure. Furthermore, a comparative analysis of the properties of the wild-type and mutant amino acids reveals a discrepancy in their size. The mutant residue, being smaller, may result in a reduction of interactions within the protein structure. This size difference could lead to the loss of stabilizing interactions, such as hydrogen bonds or van der Waals forces, that are crucial for maintaining the structural integrity and function of the protein. Therefore, this mutation could potentially lead to a destabilized protein structure, further emphasizing the potential pathogenicity of this mutation. We evaluated the pathogenicity of the *TSAG10* variant using deleteriousness- predicting software and the results are presented in Table 2. All seven-software predicted that this missense variant is deleterious. These findings provide further evidence to support the hypothesis that the p. Leu371Pro variant in *TSGA10* is likely to be pathogenic and responsible for the infertility observed in patients (III: 1) and (III: 2).

**Table 2:** Summary of the pathogenicity predictions of the *TSAG10* variant

**Gene** *TSAG10*	**Mutation position**		**Mutation type** Missense	**Exon** 15
	**cDNA** c.1112T > C	**Protein** p. L371P		
**Prediction**			**Score / % Expected accuracy**	
**SIFT**^a^				
Affect protein function			0.01	
**PolyPhen-2**^b^				
Probably damaging			1.000	
**PolyPhen-1**				
Deleterious			74%	
**PredictSNP**^c^				
Deleterious			61%	
**PANTHER**^d^				
Deleterious			74%	
**MAPP**^e^				
Deleterious			84%	
**SNAP**^f^				
Deleterious			81%	

^a^SIFT: (*Stop, Investigate, Find, Trace)* assigns scores closer to 0.00 to SNPs that are more likely to be damaging)

^b^PolyPhen-2: (Polymorphism Phenotyping) provides a score between 0 and 1, with scores closer to 1 indicating a higher confidence in predicting the SNP as damaging)

^c^PredictSNP: (Used for prediction of the effects of mutation on protein function)

^d^PANTHER: (Protein analysis through evolutionary relationships)

^e^MAPP: (Multivariate Analysis of Protein Polymorphism) A method that aims to predict the deleteriousness of non-synonymous single nucleotide polymorphisms)

^f^SNAP: (Scala Nucleotide Alignment Polymorphism)

### TEM analysis of spermatozoa

**Fig. 3:**
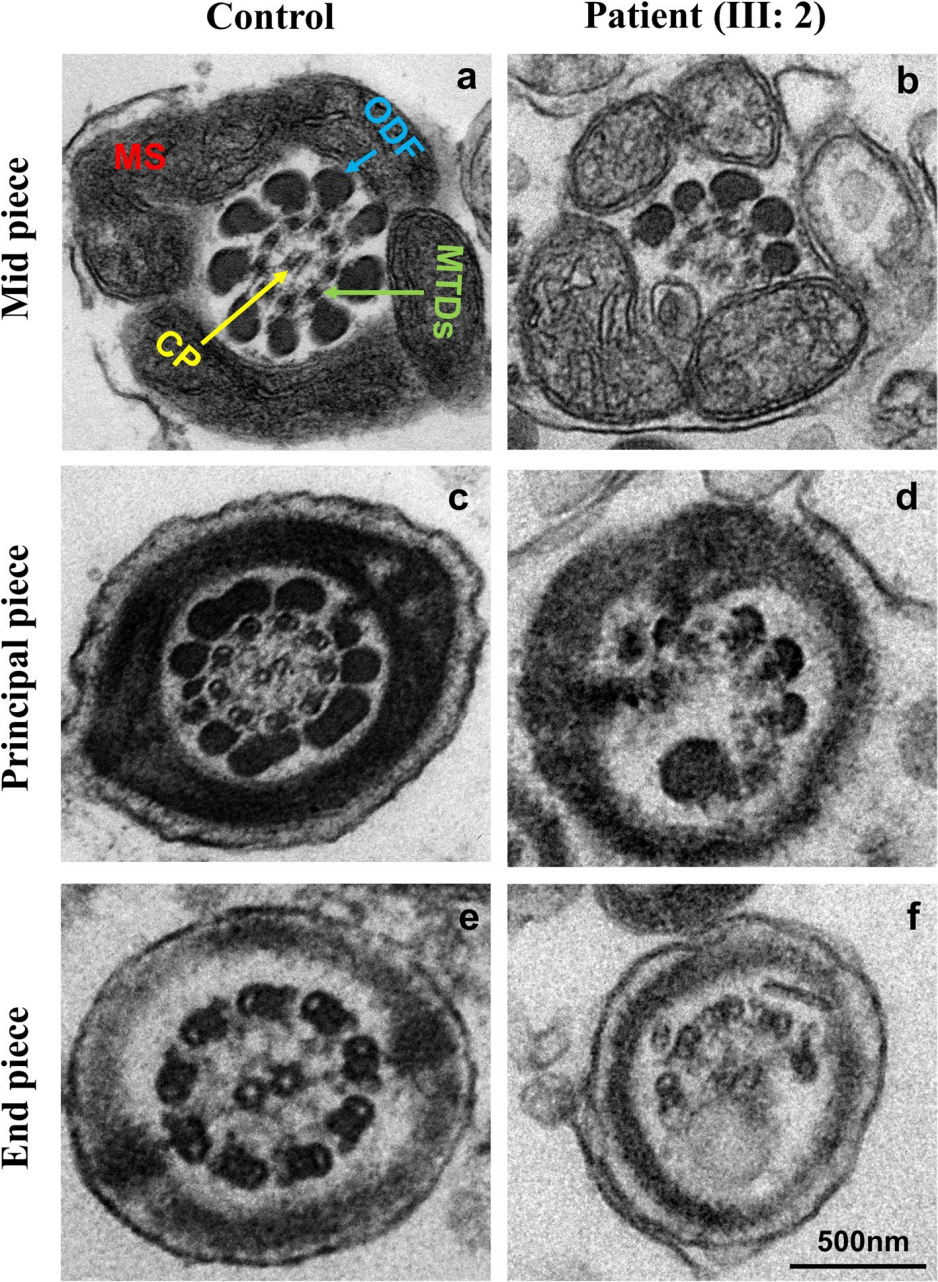
Ultrastructural defects in spermatozoa from the patient carrying homozygous *TSGA10* variant. Transmission electron microscopic morphology of the cross-sections of the midpiece, principal and end-piece in patient and normal control. Scale bar: 500 nm. Abbreviations: MS, mitochondrial sheath; CP, central pair; ODF, outer dense fiber, MTDs; microtubule doublets

TEM was carried out to check the ultrastructure of the sperm flagella of III: 2. Semen samples from a fertile male were used as a control. In contrast to the typical axoneme ultrastructure observed in sperm flagella from a control, which is composed of nine doublets of microtubules circularly arranged in an organized way (9 + 2 organization), disorganized axonemal structures with most of the cross-sections lacking the central pair (CP) and some doublets of microtubules were observed in patient (III: 2), which accounts for 98% of sperm flagella total of 30 cross-sections of mid-piece, principal and end piece were analyzed (Fig. 3). Hence, these findings indicated that patient with the missense variant in *TSGA10* displayed defects in axonemal ultrastructure with the (CP) missing.

### Level of the *TSGA10* mutant mRNA in the sperm of the mutated patient

**Fig. 4:**
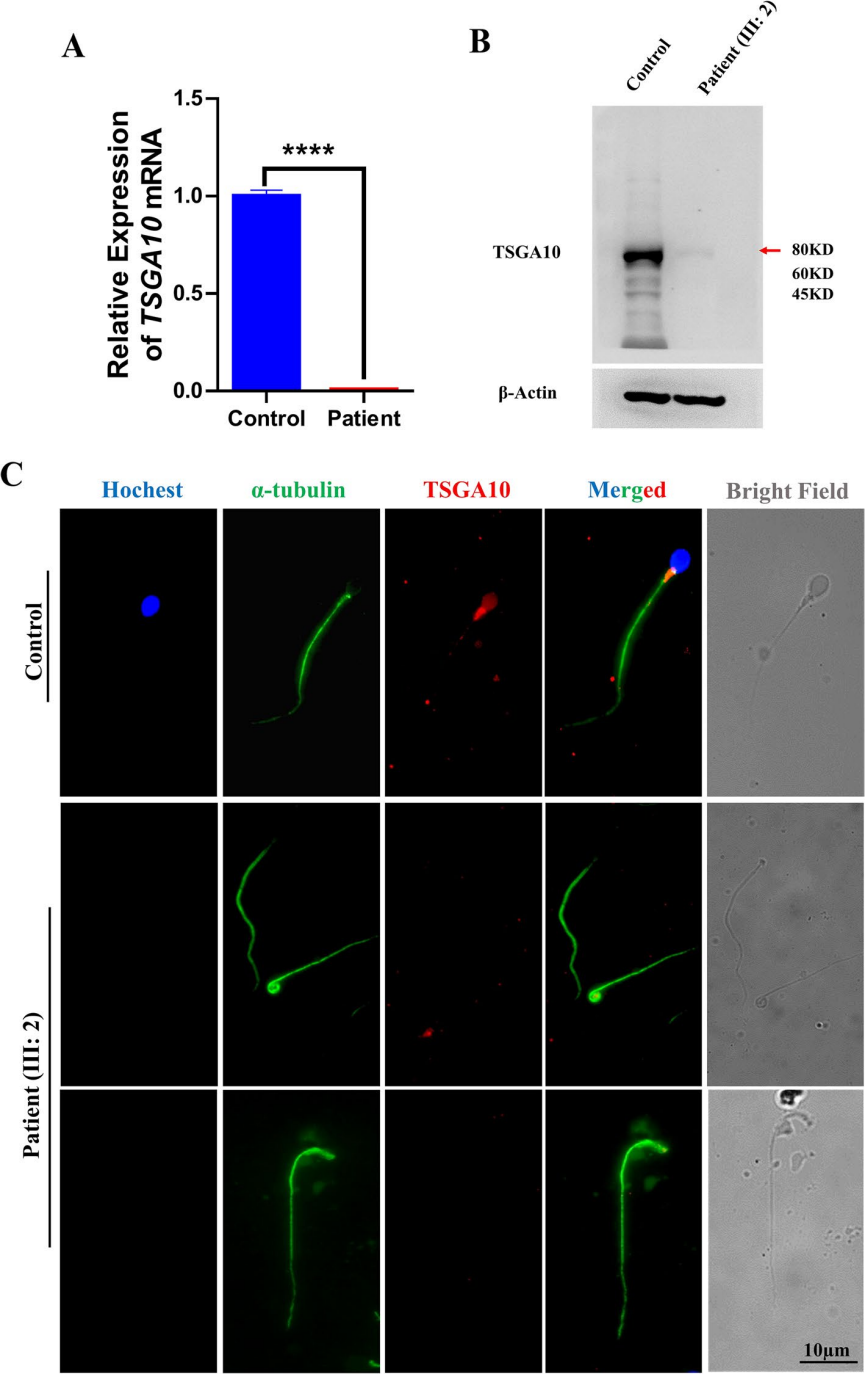
Expression of *TSGA10* in the patients’ spermatozoa. **A** Real-time quantitative PCR analysis of *TSGA10* mRNA expression in semen samples from the patient and control. β-Actin was used as an internal control. **B** Western blotting analysis of the protein level of TSGA10 in the patient and normal control. α-tubulin was used as a loading control (**C**) Representative image of spermatozoa from fertile control and patients carrying the *TSGA10* variant stained with an anti-TSGA10 antibody (red), an anti-α-tubulin antibody (green), and Hoechst (blue, nuclear marker). The TSGA10 signal was not detected in the sperm midpiece from the patient. Scale bar: 10 μm

In order to verify the effect of this novel variant on *TSGA10* expression. We performed qPCR to detect the expression level of *TSGA10* mRNA in the patient and compared to that in control samples. The qPCR analysis clearly demonstrated the presence of *TSGA10* mRNA in the control sample, while did not reveal any detectable expression of *TSGA10* in the patient semen samples (Fig. 4A), indicating that the *TSGA10* variant induce a drastic reduction of *TSGA10* mRNA levels in the sperm of the mutated patient.

### Level of the TSGA10 mutant protein in the sperm of the mutated patient’s sperm

To validate the impact of the *TSGA10* mutation on its protein expression, we performed western blot and immunofluorescence staining analysis to assess the level of the TSGA10 protein in the patient’s sperm. Western blot analysis clearly demonstrated the expression of TSGA10 in the control sample, while the TSGA10 mutant protein was hardly detectable in the patient's sperm. Furthermore, immunostaining of spermatozoa from a healthy control confirmed the localization of TSGA10 in the midpiece. In contrast, no detectable TSGA10 signals were seen in the patient’s sperm (Fig. 4B). These results further substantiated that the *TSGA10* variant, which resulted in the loss of TSGA10 protein expression, as the cause of acephalic spermatozoa in the patients (Fig. 4C).

## Discussion

TSGA10 is a testis-specifically-expressed protein, which is located to the midpiece of sperm, centrosome and basal body [14, 17]. Previous research indicated a vital function of *TSGA10* in the formation of head–tail link of centrioles, the arrangement of mitochondrial sheath and embryonic development [7, 15, 18]. So far, the genetic etiology of *TSGA10* variations have been associated with about 3.1% of reported cases suffered from acephalic spermatozoa syndrome [5, 7–13, 18, 19, 40–45].

Here we reported a homozygous missense variant in *TSGA10* (c.1112T > C, p. Leu371Pro) identified in infertile patients with acephalic spermatozoa from a Pakistani family. This variant was further verified by Sanger sequencing which revealed that the *TSGA10* variant in the mutated patients recessively co-segregating with the infertility phenotype in this family. Multiple sequence alignment interpretations suggest that the mutated Leucine amino acid is significantly conserved among species, thus predicting the deleteriousness for p. Leu371Pro variant. The *TSAG10* variant was submitted to several prediction tools to assess its degree of pathogenicity and all predicted it to be deleterious. Subsequent qPCR and western blotting revealed that the variant caused an almost complete loss of TSGA10 mRNA and protein in the sperm of the mutated patient. Moreover, the sperm phenotype of the mutated patient also resembles those of TSGA10-mutated patients previously reported [7]. Altogether, the pathogenicity of this variant is supported by in silico analysis, expression experiments, and clinical manifestations. Also, our results justify the previous reports on *TSGA10* mutant infertile patient and *Tsga10* knockout mouse model [19, 46].

We, and previous studies, observed headless sperm and defects in sperm axonemal ultrastructure, suggesting that TSGA10 plays a critical role in head/flagellum attachment, as well as sperm tail assembly and function [7]. It was reported that the C-terminus of TSGA10 was located to the midpiece of mature spermatozoa in an association with centrosome and basal body by the interaction of ODF2 protein [15, 16]. Odf2 haploinsufficiency also caused neck-midpiece separation [19], which is similar to the *Tsga10* mutant phenotype, suggesting that these two proteins might function together. In the developing axoneme, the basal body plays an important role as a nucleation site, suggesting that it could have potential roles in flagellar biogenesis during spermiogenesis. Moreover, TSGA10 was also found localizing to the developing sperm tail, suggesting that it may participating in flagellar structure directly [7]. Further investigation into the ultrastructural localizations, specific protein interactions and signaling pathways involving *TSGA10* will be critical for elucidating its precise role in sperm tail assembly and function, as well as its potential contribution to male infertility.

## Conclusion

Our study reports a novel homozygous missense variant of *TSGA10*, c.1112T > C, p. Leu371Pro, associated with ASS and male infertility in the mutated patients. This discovery represents the first identification of a missense variant in the *TSGA10* gene within a Pakistani family. This particular variant of *TSGA10* is strongly linked to the cause of acephalic spermatozoa. The findings of this study contribute new knowledge to researchers and clinicians in the field of genetics and reproduction, enhancing our understanding of the pathology and molecular mechanisms underlying ASS.

### Supplementary Information

**Additional file 1: Supplementary Figure 1.** Pipeline of the variant filtration followed by whole exome sequencing.

**Additional file 2: Supplementary Table 1.** List of primers used in this study.

**Additional file 3: Supplementary Table 2.** Information of antibodies used in this study.

## Data Availability

The authors confirm that the data supporting the findings of this study are available within the article and its supplementary materials. Additional data are available from the corresponding author upon reasonable request.

## Abbreviations

*TSGA10*: Testis specific gene 10
*ASS*: Acephalic spermatozoa syndrome
WES: Whole-exome sequencing
qPCR: Real-time quantitative polymerase chain reaction
WHO: World health organization
*SUN5*: Sad1 and UNC84 domain containing 5
*BRDT*: Bromodomain testis associated gene
*PMFBP1*: Polyamine modulated factor 1 binding protein 1
ODF2: Outer dense fiber of sperm tails 2
USTC: University of science and technology of China
PFA: Paraformaldehyde
PBS: Phosphate buffered saline
MAF: Minor allele frequencies
CP: Central pair
WT: Wild-type
MT: Mutant
