# Supplementary material for: A novel homozygous TSGA10 missense variant causes acephalic spermatozoa syndrome in a Pakistani family
Source: Basic Clin Androl. 2024 Feb 5;34:4. doi: 10.1186/s12610-024-00220-7 (PMC10840149; doi:10.1186/s12610-024-00220-7)
Supplement: Supplementary file 1 — Additional file 1: Supplementary Figure 1. Pipeline of the variant filtration followed by whole exome sequencing. [file 12610_2024_220_MOESM1_ESM.docx]

**Whole exome sequencing of gDNA from the patients (III: 1 & III: 2) & their mother (II: 1) & their fertile brother (III: 3)**

**116 variants in 91 genes**

Variants affect protein sequence

Recessive inheritance

**2 variants in 2 genes**

**3141 variants, 1410 genes**

Variants within genes predicted to be non-deleterious by >50% software covering them were omitted

Variants with MAF>0.05 in the 1000 Genomes, ESP6500 or ExAC were excluded, variants homozygous in our in-house 578 fertile men (41 Pakistanis, 254 Chinese and 283 Europeans) were excluded.

**1 variant in 1 gene**

Sanger sequencing

**Gene: *TSGA10***

**Variant: c.T1112C**

**Additional Figure 1**: Variants filtration from whole exome sequencing data

WES data analysis flow chart. For MAF>0.01, the 1000 Genomes, ESP6500, and ExAC or GenomAD databanks were browsed. In-house genomic data of 578 (41 Pakistani, 254 Chinese, and 283 Europeans) fertile men were used. WES, whole-exome sequencing; MAF, minor allele frequency.
