# Supplementary material for: A novel homozygous TSGA10 missense variant causes acephalic spermatozoa syndrome in a Pakistani family
Source: Basic Clin Androl. 2024 Feb 5;34:4. doi: 10.1186/s12610-024-00220-7 (PMC10840149; doi:10.1186/s12610-024-00220-7)
Supplement: Supplementary file 2 — Additional file 2: Supplementary Table 1. List of primers used in this study. [file 12610_2024_220_MOESM2_ESM.docx]

| ***TSGA10* Variant** | **Product size (bp)** | **Forward primer** | **Reverse primer** |
| --- | --- | --- | --- |
| **c.T1112C p. L371P** | 450 | CCCTTGGAGAGAGTTTGGCA | ATCTGCCTCTGATTGACGGG |
| **ACTB** | 130 | AATGAGCTGCGTGTGGCTC | ATAGCACAGCCTGGATAGCA |

**Supplementary Table 1.** Primers for Sanger sequencing of *TSGA10* variant

bp: base pair
