# Supplementary material for: A novel homozygous TSGA10 missense variant causes acephalic spermatozoa syndrome in a Pakistani family
Source: Basic Clin Androl. 2024 Feb 5;34:4. doi: 10.1186/s12610-024-00220-7 (PMC10840149; doi:10.1186/s12610-024-00220-7)
Supplement: Supplementary file 3 — Additional file 3: Supplementary Table 2. Information of antibodies used in this study. [file 12610_2024_220_MOESM3_ESM.docx]

**Supplementary 2 Table:** Information of antibodies used in this study

| **Primary antibodies** | | | | | | | | |  |
| --- | --- | --- | --- | --- | --- | --- | --- | --- | --- |
| **Target** | **Dilution** | | **Host species** | | **Supplier** | | **Catalog number** | |  |
| TSGA10 | 1:1000 (WB)  1:100 (IF) | | Rabbit | | Proteintech Group | | 12593-1-AP | |  |
| ACTL7A | IF 1;100, | | Rabbit | | Proteintech Group | | 17355-1-AP | |  |
| α-Tubulin | 1:100 (IF) | | Mouse | | Sigma Aldrich | | F2168 | |  |
| β-Actin | 1:2000 (WB) | | Rabbit | | Abcam | | ab8227 | |  |
| **Secondary antibodies** | | | | | | | | |  |
| **Target** | | **Dilution** | | **Host species** | | **Supplier** | | **Catalog number** | |
| Goat anti-Mouse IgG1  Alexa Flour 488 | | 1:100 (IF) | | Goat | | Invitrogen | | A21121 | |
| Donkey anti-Rabbit IgG  Alexa Flour 555 | | 1:200 (IF) | | Donkey | | Invitrogen | | A31572 | |
| Donkey anti-Rabbit IgG  HRP | | 1:10000 (WB) | | Donkey | | Bio legend | | 406401 | |

IF: Immunofluorescence, WB: Western blot
